# A scoring strategy for progression risk and rates of treatment completion in subjects with latent tuberculosis

**DOI:** 10.1371/journal.pone.0207582

**Published:** 2018-11-15

**Authors:** Michael Scolarici, Ken Dekitani, Ling Chen, Marcia Sokol-Anderson, Daniel F. Hoft, Soumya Chatterjee

**Affiliations:** 1 St Louis University School of Medicine, St Louis, MO, United States of America; 2 Division of Biostatistics, Washington University in St. Louis School of Medicine, St Louis, MO, United States of America; 3 Division of Infectious Diseases, Allergy and Immunology, Department of Internal Medicine, St Louis University, St Louis, MO, United States of America; Agencia de Salut Publica de Barcelona, SPAIN

## Abstract

It is unknown whether patients with LTBI at high vs. low risk of developing active TB are currently adequately identified and treated in the US. In this study our objective was 1) To retrospectively apply the online calculator (tstin3d.com) to determine the probability of having LTBI and assign cumulative risk of progression. 2) Measure treatment outcomes in subjects with Low: 0-<10%, Intermediate: 10-<50% and High: 50–100% cumulative risk. We performed medical record review of tuberculin skin test and/or Interferon-γ release assay (IGRAs) positive patients with LTBI seen from 2010–2015. Of 125 subjects included, 51(41%), 46 (37%) and 28 (22%) subjects were in Low, Intermediate and High risk groups respectively. Tstin3d.com was useful in determining the probability of LTBI in tuberculin skin test positive US-born subjects. Overall treatment completion rate was 61% in 114 subjects with complete treatment information and similar completion rates were seen in the three groups (Low-60%, Intermediate-63% and High-57%). Provider assessment of important clinical risk factors was often incomplete. Logistic regression analysis showed no association of assessment of important risk factors with treatment completion. The major limitations of the calculator are the lack of an updated data on country-specific prevalence of TB disease as the global burden of TB continues to decrease as well as falsely high positive predictive values that due to “transiently” positive IGRA results in subjects from countries with low prevalence. Nonetheless, our findings suggest that tstin3d.com could be utilized in the US setting for improving providing awareness of risk stratification of patients with LTBI for short course treatment regimens based on risk.

## Introduction

Treatment of Latent Tuberculosis Infection (LTBI) decreases the risk of progression to active TB by 60–90% [1]and both the CDC and WHO recommend treatment of subjects with LTBI [2] in low burden TB countries like the US where the overall active TB rates are <10/1000 population. However, TB incidence in the US has plateaued at 3.0 cases per 100,000 persons from 2013–2015 [3]. The exact reasons for this are unclear but poor rates of LTBI treatment completion (i.e. 50% or less on average) [4] is a likely contributing factor. In part, poor completion rates are due to prolonged treatment required in subjects with LTBI with Isoniazid (INH) for 6–9 months or 4 months of Rifampin. Recently, a 3 month directly observed regimen of weekly INH and Rifapentine (3HP) is approved by the CDC, has shown equivalent efficacy. This regimen is only recommended in select clinical high risk groups [5]. Although certain clinical risk factors such as being infected with HIV, diabetes, recent contact with an active TB patient or receiving immune modulatory drugs like TNF-α blocker therapy are well known to increase the risk of developing TB disease, data are lacking on the frequency with which a comprehensive assessment of all clinical risk factors is performed in subjects with LTBI by health care providers. Provider awareness of a subject being at high risk of progression to active TB could facilitate treatment completion using shorter regimens in those at high risk. www.tstin3d.com is a free online calculator that combines TST and/or IGRA screening results with other clinically pertinent information, to estimate the positive predictive value (PPV) of TB infection as well as individual’s annual and cumulative risk of progression to active TB. The calculator has never been externally validated as a study looking at progression of LTBI to active TB will be unethical in the US and current CDC guidelines recommend offering treatment to all subjects with LTBI.

Such assessments not only allow the identification of patients that are at high risk of progression [6] but could be used to select shorter, supervised regimens to ensure treatment completion in that group. Therefore, to assess utility of the calculator in a clinical setting, we used it to perform a retrospective systematic quantification of risk, assessed provider risk awareness and compared treatment completion rates in subjects at “Low”, “Intermediate” and “High” risk of LTBI reactivation.

## Study population and methods

Data were collected retrospectively on 125 adult patients (≥18 years and ≤80 years) with LTBI (screened using ICD9/10 LTBI diagnosis codes) that were seen in the Saint Louis University Infectious Diseases outpatient clinic between January 1, 2010 and December 31^st^, 2015. Patients with a positive TST and/or IGRA result without active TB or prior history of treatment for LTBI or active TB were included. Variables (listed in **Supplemental Methods)** required by tstin3d.com to estimate an individual’s annual and cumulative risk of LTBI reactivation were obtained from patient electronic medical records. Data on race was collected only for US born individuals. Information about type and duration of antibiotic therapy, last known documented follow-up, frequency with which healthcare providers assessed the risk factors affecting risk of progression to active TB and reasons for discontinuation were obtained. The study was approved by the St Louis University Institutional Review Board which waived the requirement for Informed consent as de-identified patient information was used.

### Variables generated by the calculator

The calculator generated positive predictive value (PPV), annual and cumulative risks (up to age 80 years) of progression to active TB disease (details in **Supplemental Methods)**. The calculator was developed from comprehensive data obtained from systematic reviews and metaanalysis [7, 8] and encompasses all the well-known risk factors predisposing to TB disease. It derives the baseline annual risk of TB disease from a large cohort (1,216,425 subjects) of healthy TST-positive US military recruits followed up for 4 years to study development of TB disease [9]. The cumulative risk refers to the annual risk of TB reactivation multiplied by the number of years before the patient reaches an age of 80 years.

### Analysis

Concordance between TST and IGRA in subjects that received both tests was analyzed by McNemar’s test of corrected proportions. Descriptive statistics were analyzed to determine the characteristics of subjects with a PPV of greater than 50% along with characteristics and treatment outcomes of patients in Low (<10%), Intermediate (10% to <50%), and High (50%-100%) cumulative risk categories. Fisher’s exact test was used to determine the association between the types of drug regimen with treatment completion rates. Logistic regression analysis was performed to examine the association between provider assessment of an individual clinical risk factor and the patient’s likelihood of completing the treatment. All risk factor assessments were done per available documentation in patient’s electronic medical record, either from the Infectious Diseases clinic provider notes or notes entered by any healthcare provider. If data on a specific medical risk factor was not available, it was decided to not include it for cumulative risk assessment. If documentation of BCG status was not available in the medical record, we used the patient’s country of origin to determine BCG vaccination policy per the BCG world atlas (http://www.bcgatlas.org/index.php). All statistical analyses were conducted using SAS 9.4 (SAS Institute, Cary, NC), two-sided with a significance level of 0.05.

## Results

### Characteristics of the study subjects

**Fig 1:**
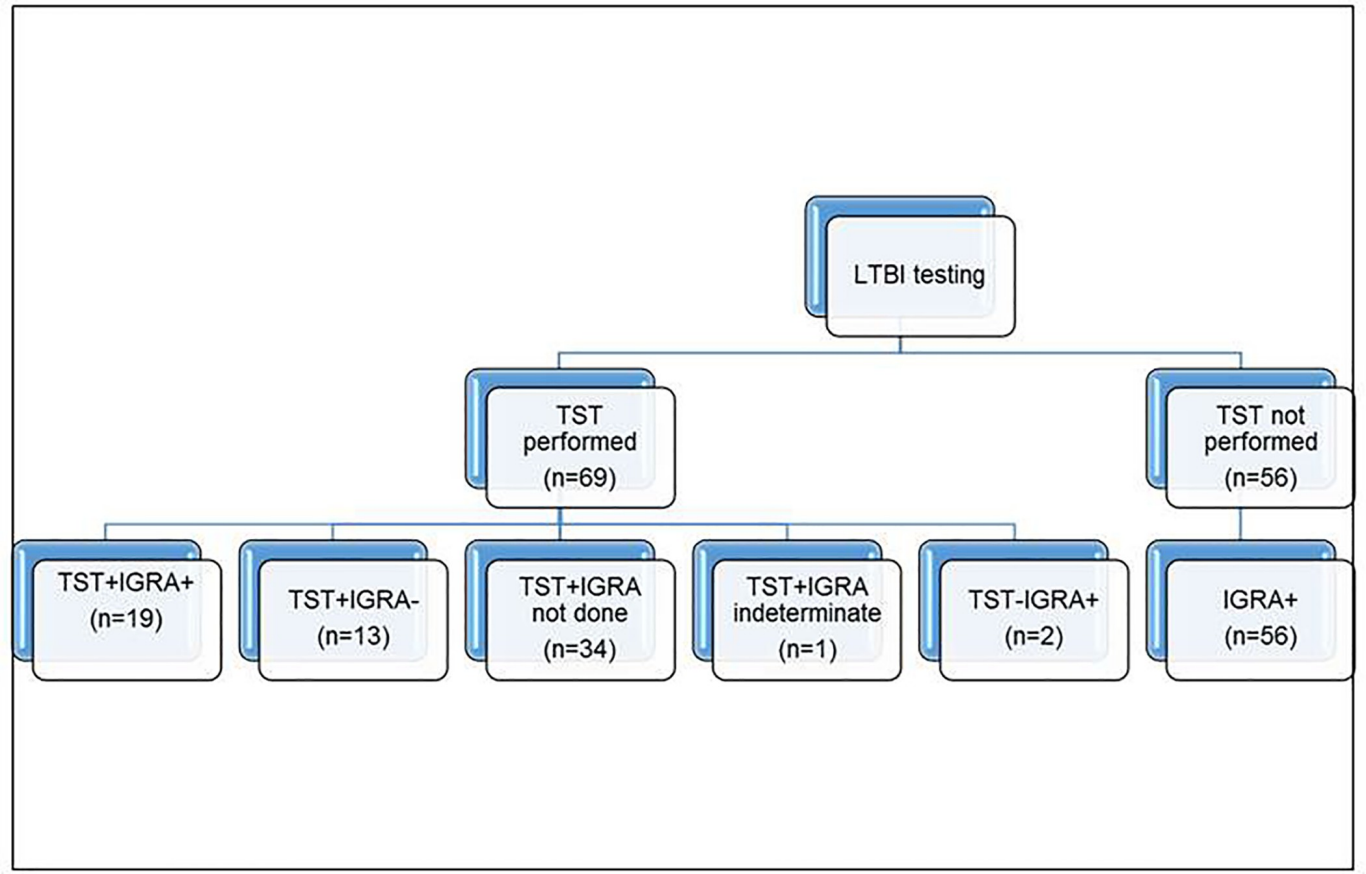
Showing all subjects included in the study based on whether they had a tuberculin skin test (TST) performed. Those who tested negative on TST or had no TST results available had to have a positive Interferon Gamma Release Assay (IGRA) i.e. Quantiferon-TB Gold or T-spot TB test to be included in the study.

**Table 1** shows the baseline demographic information for the study subjects. The median age was 49 years with 43.31% female. Of the 125 patients included, 94 were from the US and US territories, 30 were non-US born and 1 unknown. Among US-born, most were Caucasian (52%) or African American (43%). The mean age at immigration for non-US born subjects was 28.5 years. TST data were available on 69 and IGRA data on 91 subjects. Of all 35 TST positive subjects with IGRA results available, 19 were IGRA positive, 13 were negative and 1 indeterminate. Only 2 subjects with positive IGRA had a negative TST result (**Fig 1**). There was significant discordance between the two tests (p = 0.0045, McNemar’s test) in subjects who underwent both TST and IGRA for diagnosis. 56 had a positive IGRA (either a Quantiferon-TB Gold or T-spot TB) and 34 had a positive TST as their only test.

**Table 1:** Characteristics of patients with latent tuberculosis infection.

Patient Characteristics		Number (min to max)
Median Age (years)		49, Range:22–80
Median age at immigration to USA (years)		28.5 (3–45)
Height (cm)		170.2 (147.3–195.6)
Weight (kg)		82.5 (47.3–175.1)
BMI (kg/m^2)		29.15 (18.7–55.39)
**Patient demographics**		**Number (%)**
Sex (Female)		54 (43.2%)
Ethnicity		
Caucasian		48 (38.4)
African American/Black		39(31.2)
Hispanic		1
Other		35(28)
Unknown		2(1.6)
**Chest X-ray**		
No abnormalities		86
Fibronodular disease		9
Suspected granuloma		10
Other		20
**Country of Birth**		
** **	Bosnia	4
	Burma/Myanmar	1
	Congo	2
	India	3
	Iraq	2
	Ireland	1
	Kenya	1
	Malaysia	1
	Mexico	1
	Nepal	3
	Pakistan	2
	Philippines	1
	Russia	1
	Somalia	2
	Thailand	1
	USA	94
	USA-Puerto Rico	1
	Vietnam	3
	Unknown	1
Total		125

### Positive predictive value of TST

Foreign born subjects who were TST positive/IGRA unavailable were from high or intermediate TB burden countries and consequently had a PPV value of 50% or greater for being TST positive. Therefore, tstin3d.com was primarily useful for calculating the PPV in 22 US-born subjects with positive TST with indeterminate or unavailable IGRA. The calculator assigns higher PPV to the Non-Hispanic Black/African-Americans with a positive TST and consequently higher number of Non-Hispanic Black/African-American subjects were in the >50% PPV group compared to those with PPV<50%.

### Risk of reactivation

**Fig 2:**
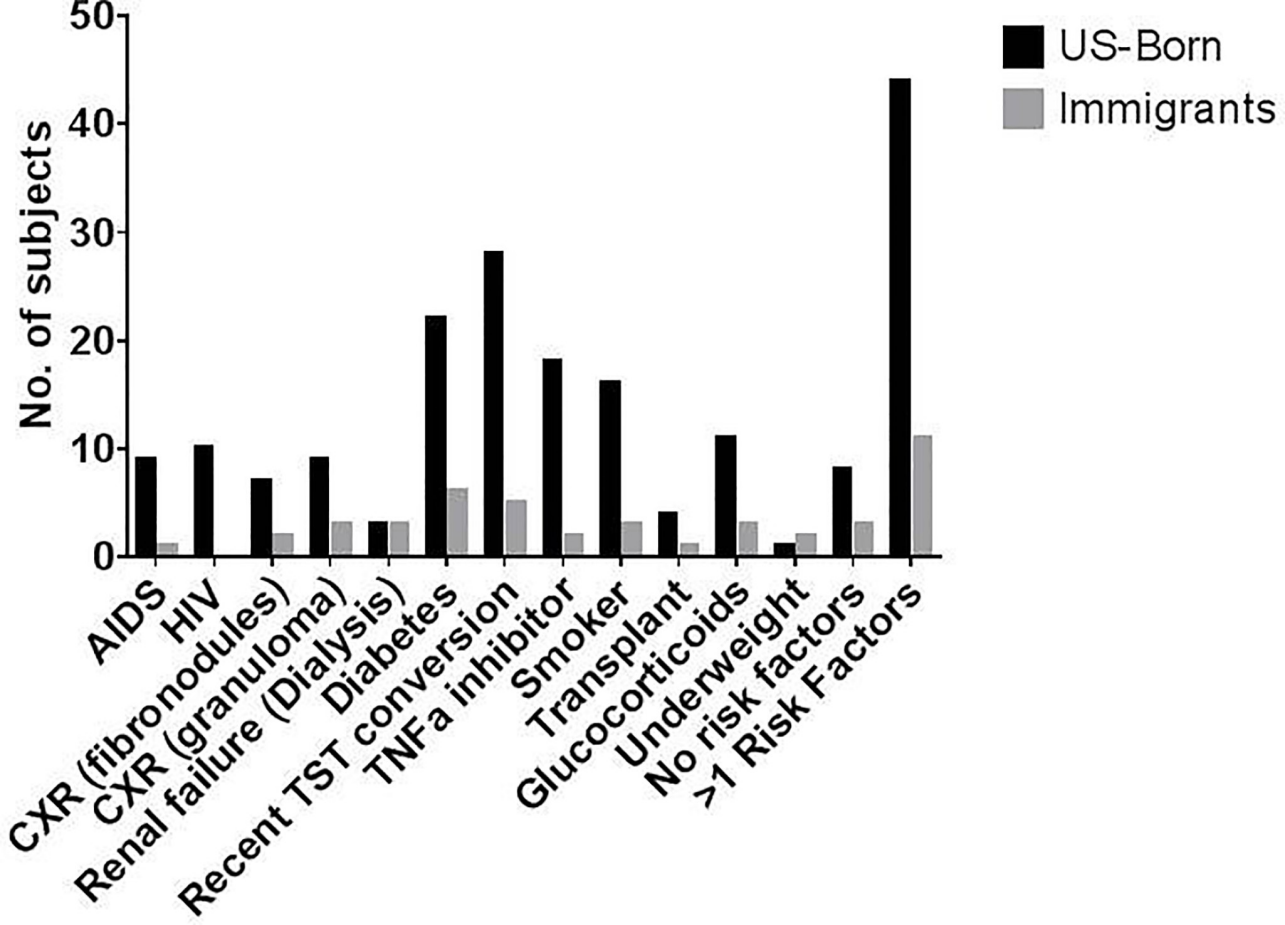
Showing number of US born (black bars) vs. non-US born (grey bars) subjects with different risk factors for progression to active TB disease in the overall cohort of patients with latent TB.

The overall distribution of risk factors in the study patients comparing US born with immigrants is shown in **Fig 2.** HIV, diabetes, history of smoking, taking a TNF-α inhibitor and on transplant immunosuppression were more common in US born subjects compared to immigrants. The median cumulative risk in TST positive/IGRA negative individuals was 19%, suggesting that they were not in the high-risk group. AIDS patients had the highest annual (>10%) and cumulative (>50%) risk. A larger proportion of African Americans (20% and 43%) were in the highest annual and cumulative risk category compared to Caucasians (2% and 16%) and this could be partly due to higher risk assigned to African Americans compared to age and sex matched Caucasians. However, the risk increase was primarily because of the significantly increased prevalence of HIV/AIDS noted in African Americans (15/42, 37.5%) compared to Caucasians (4/49, 8.2%) (p = 0.001 Fisher exact test). **Table 2** shows characteristics of all the patients in the High cumulative risk group.

**Table 2:** Distribution of medical risk factors for progression to active TB in subjects in the High (50–100% cumulative risk) group.

Risk factor	Number	Cumulative risk value%
**HIV (non-AIDS)**	10	100 (Median risk) *
• No comorbidities	0	
• Smoker	2	
• Recent TST conversion (≤ 2 years ago)	9	
**AIDS**	10	100 (Median risk) *
• No comorbidities	2	
• Fibronodular disease on chest X-ray +Recent TST conversion (≤ 2 years ago) +renal failure on dialysis	1	
• Smoker	4	
• Smoker+ Recent TST conversion (≤ 2 years ago)	2	
• Recent TST conversion (≤ 2 years ago)	6	
• Diabetes	1	
Recent TST conversion (≤ 2 years ago) +renal failure on dialysis	1	85.04
Renal failure on dialysis	1	70.3
Recent TST conversion + TNF-α blocker + steroid use	2	51.69, 8.61
Immunosuppression after solid organ transplant	2	100, 87.51
Diabetes+ steroid use + solid organ transplant	1	100
Fibronodular disease on chest X-ray + solid organ transplant	1	69.97
Fibronodular disease on chest X-ray + bone marrow transplant	1	76.15

*All subjects with HIV and/or AIDS had 100% cumulative risk

### Risk factors and treatment completion in specific high risk groups

#### Subjects on TNF-α blockers and LTBI

Data were available for 20 subjects with LTBI on TNFα blockers. A significantly higher proportion (75%) of subjects on TNFα blockers (TNFα group) were seen because of recent (< 2 years ago) TST/IGRA conversion, compared to 17.5% in the control group (76 HIV-negative subjects not on any immunosuppressive therapy, p = 0.002, Fisher exact test). Subjects on TNFα blockers had less diabetes (6% vs. 26%), smoking >1 pack per day (6% vs. 11%), and renal failure (0% vs. 6%) compared to the control group. The median cumulative risk of progression to active TB was 17.25% in the TNFα group (range: 8.6–51.7%) and 5.6% in the control group (range: 0.1–100%). 70% of patients were able to successfully complete LTBI therapy in the TNFα group.

#### Subjects with HIV and LTBI

Of the HIV positive population with LTBI, 10 of 20 had AIDS. The median annual TB-risk amongst HIV, AIDS, and HIV-uninfected patients was 8% (3–8%), 22% (21–25%), and 0.5% (0–6%). More patients with HIV/AIDS smoked >1 pack-per-day (30% vs. 13%) compared to HIV-uninfected. Other risk-factors distributed amongst HIV, AIDS and HIV-uninfected groups were recent TST/IGRA conversion (10%, 30%, and 28%), renal-failure requiring hemodialysis (0%, 10%, and 0%), and diabetes (0%, 10%, 25%). Most HIV positive patients were prescribed INH (HIV/AIDS: 85%, HIV-uninfected: 39%). The median treatment-days were longer (274 vs. 117 days) but the rates of treatment completion were comparable for HIV/AIDS than HIV-uninfected patients (30% vs. 34%). Within the HIV positive population, there was no difference in CD4+ T cell counts between those that completed LTBI treatment vs. those that did not. Reasons HIV/AIDS patients stopped therapy included loss to follow-up (2/6), cost (1/6), hepatitis (1/6), rash (1/6), and clinical contraindications (1/6).

#### Treatment completion

**Fig 3:**
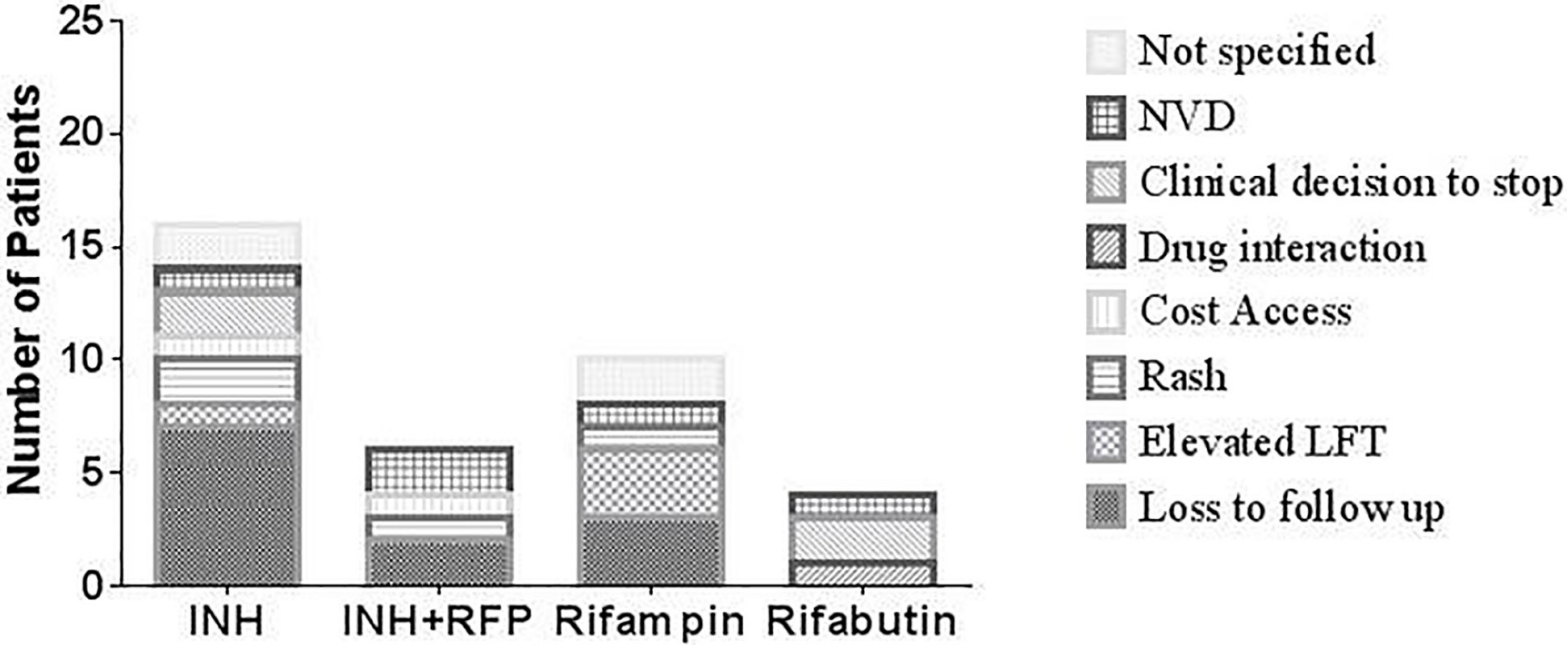
Showing the number of patients discontinuing therapy and the primary documented causes by drug group.

Of the 125 subjects included in the analysis, 8 were not offered any treatment and 3 refused. 59 of 114 (52%) subjects were started on treatment with INH, 24 (21%) on 3HP, 23 (20%) on Rifampin and 8 (7%) on Rifabutin. 69 (61%) subjects completed the recommended duration of therapy during the study period and none developed active TB. The completion rates of those treated for LTBI were the best for 3HP and worst for Rifabutin, 75% and 50% subjects respectively (**S1 Table**). Amongst the documented reasons for interrupted treatment (**Fig 3**), loss to follow-up accounted for a majority (23 subjects). Other reasons for stopping that were categorized were: elevated liver enzymes, cost/access, minor side effects (nausea, vomiting, and diarrhea), rash, drug interactions and clinical decision to stop. No reasons for stopping were documented in 4 subjects. Importantly, no significant differences were observed between patients completing therapy in the Low, Intermediate and High cumulative risk groups (p = 0.54, Chi-square test).

#### Provider risk assessment

BCG status was documented for only 15 of 32 immigrants. Less than half of the patients’ initial encounter included documented information about country of birth, HIV/AIDS status, recent TST/IGRA conversion, young age at infection, recent TST/IGRA conversion (≤2 years ago) and history of cancer **(Table 3**). Most patients were assessed for smoking and malnutrition and had received a chest X-ray at or within 3 months of their Infectious Diseases clinic visit. As shown in **Table 4**, logistic regression analysis showed that there was no statistically significant association of risk factor assessment in the clinic at initial visit with the probability of subjects completing treatment. However, we noted higher odds of treatment completion in those with documentation of immunosuppression (HIV/AIDS, steroids, transplant related immunosuppression) as well as of smoking and recent (≤ 2 years) TST/IGRA conversion.

**Table 3:** Subjects with latent tuberculosis who were assessed for the different risk factors by infectious diseases clinic providers.

Risk factor	Number of Patients Specifically Assessed in Clinic (%)
QTST	74/125 (59%)
QIGRA	92/125 (74%)
Qage	124/125 (99%)
Qimmigration	30/125 (24%)
QBCG (for immigrants)	15/32 (47%)
QCountryBirth	41/125 (33%)
QTB Contact	64/125 (51%)
QAIDS	24/125 (19%)
QHIV	45/125 (36%)
QCXR	117/125 (94%)
QRenal Failure	90/125 (72%)
QDM	43/125 (34%)
QRecent PPD/IGRA conversion	58/125 (46%)
QSilicosis	0/125 (0%)
QTNFa	37/125 (30%)
QYoungTB	1/125 (0.8%)

**Table 4:** Logistic regression analysis of relating probability of completion of treatment to assessment of selected individual TB progression risk factors.

Assessed Risk factor*	Odds Ratio (Confidence Interval)	p value
Renal failure	0.83 (0.35–2.0)	0.67
HIV	1.83 (0.83–4)	0.13
AIDS	1.36 (0.53–3.5)	0.52
Chest X-ray available	0.36 (0.082–1.6)	0.18
Diabetes mellitus	0.89 (0.4–1.9)	0.75
Recent TST conversion (≤2 years ago)	0.78 (0.37–1.7)	0.51
On TNF-α blocker	0.72 (0.31–1.7)	0.45
Smoker	1.2 (0.43–3.4)	0.72
On steroids	0.99 (0.39–2.5)	0.97
Transplant immunosuppression	1.56 (0.2–11.4)	0.67

*Young age at TB and Head and Neck cancer are not included as number of subjects assessed were not enough to include in logistic regression analysis

## Discussion

Our study demonstrates that tstin3d.com can be used to divide subjects with LTBI into Low, Intermediate and High cumulative risk of developing active TB based on the presence or absence of a combination of clinical risk factors in the US setting. Current standard of care practice did not result in higher treatment completion rates for patients in the High-risk group-an important goal of TB control in low burden countries. Additionally, evaluation of a particular risk factor was not associated with improved treatment completion but this could be because providers often select regimens based on side effect profiles of drugs rather than the patient’s risk of progression to active TB.

For patients undergoing TST, the calculator (tstin3d.com) facilitates risk interpretation by using the TST size, PPV of TST and risk of development of disease. A PPV >50% leads to an increased cumulative risk. There are several limitations of using tstin3d.com, the primary ones being that it is based on 2004 WHO estimates of country-specific smear positive TB disease rates and utilizes older data on risk based on TST. Furthermore, the calculator may underestimate the effect of non-tuberculous mycobacterial infections which tend to increase as the prevalence of tuberculosis decreases in a country [10]. Additionally, the effects on TST reactions of BCG and NTM are derived from studies in non-HIV-infected persons, so may not be accurate in HIV-infected persons. Furthermore, the calculator assigns a default PPV>98% to those with a positive IGRA. False positive IGRAs remain a concern among specific groups assessed for LTBI in countries with low prevalence [11]. Similar to others, we found significant discordance between PPD and IGRA positivity in our study [12–14]. We found the calculated PPV to be most useful in stratifying TST positive US born subjects with unavailable IGRAs as they are not vaccinated with BCG. Thus, as the global burden of TB decreases, physicians treating patients with LTBI should consider updated data on country-specific prevalence of TB disease. Furthermore, they should be mindful of a falsely high PPV that can be assigned by tstin3d.com due to “transiently” positive IGRA results in subjects from countries with low prevalence e.g. US healthcare workers.

We noted equivalent treatment completion rates between the different risk groups. Current CDC guidelines define specific risk groups which should be given high priority for LTBI treatment based on TST size and/or IGRA positivity and clinical risk factors [14]. However, as shown in our study, these risk factors may not be systematically assessed at all times by the busy clinician. Although lack of adequate healthcare as well as patient related factors e.g. homelessness, affect adherence to LTBI treatment [4, 15, 16], specific interventions like shorter duration of therapy using Rifamycin based regimens and directly observed treatment (DOT) are well recognized measures to improve treatment adherence [17–19]. This approach also minimizes the loss to follow up which was the major reason for incomplete treatment in our cohort. Since this was a retrospective study with a relative small sample size we did not find any associations of loss to follow-up with specific clinical or social characteristic (e.g. homelessness, lack of social support) as reported previously [4].

In agreement with the published literature, we found the highest rates of treatment completion in the 3HP group. Cytochrome P (CYP) 450 isoenzyme induction by Rifamycin based regimens remains a concern in subjects with HIV on ARVs. This is especially important as more than 85% of subjects with HIV/AIDS were prescribed INH and only 30% were able to complete treatment in our study. Current IDSA guidelines recommend use of 3HP only with efavirenz (EFV)—or raltegravir (RAL)-based regimens (in combination with either abacavir/lamivudine [ABC/3TC] or tenofovir disoproxil fumarate/emtricitabine [TDF/FTC]) for treatment of LTBI [5]. A future strategy of temporarily switching HIV positive patients to the above-mentioned regimens, if clinically feasible, could allow better treatment completion rates with a 3HP based regimen.

Subjects on TNF-α blockers were referred mainly because of recent TST/IGRA conversion (within ≤ 2years) which increases their risk of TB reactivation disease. Rifamycin based regimens have recently been shown to be effective with minimal side effects in this group of patients [20]. Although only 25% of patients on TNF-α blockers were prescribed 3HP, 70% were able to successfully complete LTBI therapy. This is likely due to close clinic follow-up that these patients receive for their underlying autoimmune disease.

An important limitation of our study is the retrospective nature of data collection from the electronic health records. If data on a specific medical risk factor was not available, it was decided to not include it for cumulative risk assessment which could have led to underestimation of actual risk. A second limitation was that subjects already receiving care for co-morbid conditions at different clinics were referred to our Infectious Diseases clinic, making them more likely to seek healthcare. These subjects are therefore more likely to have higher rates of LTBI treatment completion compared to the overall population of subjects with LTBI. Furthermore, as patients were often referred from community and other specialty clinics, ID clinic providers were often aware of medical comorbidities for e.g. HIV/AIDS, Diabetes, renal failure on dialysis, and ongoing use of TNF-α blocker use at initial clinic assessment. Therefore, we probably overestimated the actual frequency with which these risk factors would be assessed had the provider not been made aware beforehand. Nevertheless, we used relatively strict criteria for inclusion of subjects with previously untreated LTBI in our study and had follow up data on the majority of our study cohort. Since no numerical risk assessment is performed routinely by healthcare providers treating patients with LTBI, our study suggests that tstin3d.com could be a useful tool for improving provider awareness of patients at higher risk of developing TB disease. Furthermore, prospective design would also allow for testing whether treatment completion rates can be improved by “risk score targeted” treatment (i.e. selecting a 3HP based regimen for all subjects at high cumulative risk). The utility of “risk score targeted” treatment as a strategy for decreasing the community burden of TB in the US needs to be validated in future prospective studies.

## Supporting information

S1 File Supplemental methods. (DOCX)

S1 Table Subjects completing treatment by drug category. (DOCX)

S2 Table Subjects completing treatment for latent tuberculosis divided by risk group. (DOCX)
