# Supplementary material for: A scoring strategy for progression risk and rates of treatment completion in subjects with latent tuberculosis
Source: PLoS One. 2018 Nov 15;13(11):e0207582. doi: 10.1371/journal.pone.0207582 (PMC6237398; doi:10.1371/journal.pone.0207582)
Supplement: S1 File — (DOCX) [file pone.0207582.s001.docx]

**Supplemental Methods:**

**Variables required by the calculator (TSTin3D.com) to estimate an individual’s annual and cumulative risk of progressing to active pulmonary TB (valid only for patient’s age 18-80 years of age)[1]**

- TST size
- IGRA result
- Age
- Age at immigration (if person immigrated from a high TB burden country)
- Country of birth
- Bacille Calmette-Guerin (BCG) vaccination status
- Recent contact with active TB
- Patient-specific characteristics
  - Presence/absence of HIV/AIDS
  - Abnormal chest x-ray (fibronodular disease or granuloma)
  - Chronic renal failure requiring hemodialysis
  - Diabetes mellitus (all types)
  - Recent TB infection (TST conversion <= 2 years ago)
  - Silicosis
  - Current use of Tumor Necrosis Factor (TNF)-alpha inhibitors (e.g. Infliximab/Etanercept)
  - Young age when infected (0-4 years)
  - Carcinoma of head and neck
  - Cigarette smoking (>1 pack/day)
  - Transplantation requiring immune-suppressant therapy
  - Treatment with glucocorticoids
  - Underweight (< 90 percent ideal body weight or body mass index (BMI) <= 20)

**Instructions for using the online tool**

- If the patient is born in the U.S., then an option for state and race/ethnicity appears. Race/ethnicity option only applies to US-born patients.
- If the patient is born in Canada, then an option for native/aboriginal/Indian status appears.
- If the patient is born in India, then an option for state/territory appears.
- If the patient is born in China, then an option for Province/SAR/AR/Municipality appears.
- If the patient is born in Argentina, then an option for Provinces/District appears.
- If the patient is born in Brazil, then an option for state appears.
- If the patient is born in Australia, then an option for state/territory appears.
- If the patient immigrated to a low TB incidence country (such as Canada, US, UK, Western Europe, Australia), then age at immigration is used in the calculations.

**Details of calculations**

*Positive Predictive Value (PPV)*

- The positive predictive value of the TST (PPV_TST_) is the patient’s probability of having true latent TB infection. This is determined by the likelihood of TB infection (pLTBI) and the probability of a false positive TST test (FP-TST_BCG_ + FP-TST_NTM_):


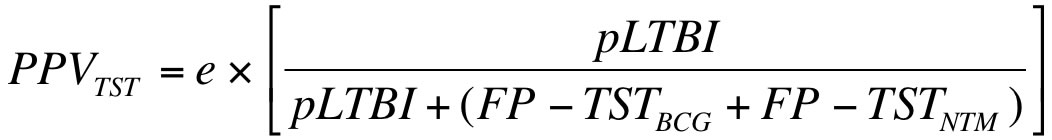


- *e* = the relative risk of a positive TST test based on the patient’s ethnic status, but only if the patient is Canadian or US-born. If the patient is not US-born, then *e* = 1. If the patient is US-born and White/Caucasian, then *e* = 1. If the patient is US-born and Non-Hispanic Black/African-American, then *e* = 3.09. If the patient is US-born and Hispanic, then *e* = 3.66. If the patient is US-born and not classified by any of the previously mentioned races/ethnicities, then *e* = 1. If patient is Canadian born and non-aboriginal then *e* = 1. If the patient is Canadian-born and aboriginal then *e* = 1.55.
- pLTBI is based on the patient’s age, the patient’s contact status (*c*), and the annual risk of infection (ARI) in the country.


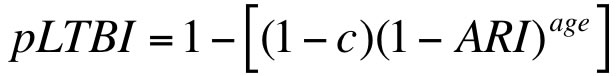


- Based on Styblo’s formula, the ARI is calculated as the incidence of smear-positive pulmonary TB per 100 000 population divided by 49. The country’s incidence of smear-positive TB in 2007 was taken from *WHO report 2009: Global tuberculosis control - epidemiology, strategy, financing* (<http://www.who.int/tb/publications/global_report/2009/en/index.html>).
- If the person tested is not a recent contact, then *c* = 0; if the person tested is a recent casual contact, then *c* = 8%; if the person tested is a recent close contact, then *c*= 40%.
- The probability of a false positive TST depends on the patient’s BCG-vaccination status (FP-TST_BCG_) and the country’s prevalence of non-tuberculous mycobacteria (NTM) infections (FP-TST_NTM_).
- If the patient is not BCG-vaccinated, then FP-TST_BCG_= 0
- If the patient was BCG-vaccinated before 2 years of age and had a TST of size…
  - 5-9 mm, then FP-TST_BCG_ = 4.2%
  - 10-14 mm, then FP-TST_BCG_ = 5.3%
  - 15+ mm, then FP-TST_BCG_ = 2.8%
- If the patient was BCG-vaccinated at 2 years of age or greater and had a TST of size…
  - 5-9 mm, then FP-TST_BCG_= 25.8%
  - 10-14 mm, then FP-TST_BCG_ = 8.7%
  - 15+ mm, then FP-TST_BCG_ = 7.8%
- FP-TST_NTM_ varies depending on the patient’s TST size and the patient’s country’s climate, which was determined using the Koppen-Geiger climate classification map, (http://koeppen-geiger.vu-wien.ac.at).
- Assumptions on IGRA test results
  - If IGRA is not done or if IGRA is negative, then PPV = PPV_TST_ (IGRA is ignored).
  - If IGRA is negative and TST is not done, then PPV = 0.
  - If IGRA is positive and TST is not done, then PPV = PPV_IGRA_ = 0.98.
  - If IGRA is positive and TST is done, then PPV = 1- ((1-PPV_TST_) x (1-PPV_IGRA_))

***Annual Risk of Disease(ARD)***

- *ARD =  PPV x [baseline annual risk of TB] x [RR for specific risk factors]*.
  - Baseline annual risk of TB = 0.1% in healthy persons with normal CXR and no other risk factors. This was taken from a large cohort of healthy TST-positive US military recruits followed up for 4 years (Comstock et al).
  - If the patient is a recent close contact, then the baseline risk of TB is 5% for the first two years and the baseline annual risk of TB is 0.1% for the years after.
    - Close contact is defined as someone living in the same household, or a non-household member who regularly (more than 4 hours per week) has contact with the index case; and is being seen at the time of recent close contact, i.e. as part of a contact investigation, but not if the person has had close contact in the remote past.
  - If the patient was recently infected, then the relative risk increases by 15 folds for the first two years, but not for the years after.
  - RR = Relative risk for specific factors - ranges from 2 to 50. See Table 2 from Chapter 4 (page 65) from 2007 Canadian TB Standards (6th edition) for values for RR.In this algorithm the highest annual risk is assigned (in descending order) to transplantation requiring immunosuppressant therapy (7.4%), HIV (5%), pulmonary silicosis (3%), chronic renal failure (requiring hemodialysis) (2.5%), carcinoma of the head and neck(1.6%), close contact of person with active TB (1.5%) and recent TST/IGRA conversion (≤ 2years) (1.5%) .

***Cumulative Risk of Disease***

- The cumulative risk of disease (CRD) is the product of the annual ARD and the number of years before the patient reaches the age of 80.

***Risk of Drug-Induced Hepatitis***

Estimates of occurrence of INH hepatitis are based on receiving INH for at least 6 months. These estimates come from 3 studies listed in the references. Risk of INH hepatitis will increase if there is concomitant viral hepatitis or other liver disease, or with alcohol consumption, particular with daily consumption. Estimates of drug induced hepatitis not available for other LTBI treatment regimens

**Reference:**

1. Menzies, D., et al., *Thinking in three dimensions: a web-based algorithm to aid the interpretation of tuberculin skin test results.* Int J Tuberc Lung Dis, 2008. **12**(5): p. 498-505.
