# Supplementary material for: A scoring strategy for progression risk and rates of treatment completion in subjects with latent tuberculosis
Source: PLoS One. 2018 Nov 15;13(11):e0207582. doi: 10.1371/journal.pone.0207582 (PMC6237398; doi:10.1371/journal.pone.0207582)
Supplement: S1 Table — (DOCX) [file pone.0207582.s002.docx]

**Supplemental Table 1. Subjects completing treatment by drug category**

| **Drug Regimen** | **Total number, n** | **Completed treatment, n (%)** |
| --- | --- | --- |
| INH | 59 | 34 (58) |
| 3HP | 24 | 18 (75) |
| Rifampin | 23 | 13 (57) |
| Rifabutin | 8 | 4 (50) |
