# Supplementary material for: A scoring strategy for progression risk and rates of treatment completion in subjects with latent tuberculosis
Source: PLoS One. 2018 Nov 15;13(11):e0207582. doi: 10.1371/journal.pone.0207582 (PMC6237398; doi:10.1371/journal.pone.0207582)
Supplement: S2 Table — (DOCX) [file pone.0207582.s003.docx]

**Supplemental Table 2. Subjects completing treatment for latent tuberculosis divided by risk group**

| **Cumulative risk group (RG)** | **Total number, n** | **Number started on treatment, n** | **Completed Treatment, n (% of those started)** |
| --- | --- | --- | --- |
| Low (0 - <10%) | 51 | 45 | 27 (60) |
| Intermediate (10 - <50%) | 46 | 41 | 26 (63) |
| High (50 - 100%) | 28 | 28 | 16 (57) |
